# Perceptions of CAH self-management and stress dosing in relation to health-related quality of life as measured by CAHQL

**DOI:** 10.3389/fendo.2026.1810137

**Published:** 2026-05-25

**Authors:** Myrto Eleni Flokas, Sarah Kulkarni, Peyton Lee, Li Yang, Charles Sukin, Sarah Kollender, Deborah P. Merke

**Affiliations:** 1 Division of Endocrinology, Children’s National Hospital, Washington, DC, United States; 2 The Eunice Kennedy Shriver National Institute of Child Health and Human Development, Bethesda, MD, United States; 3 Department of Pediatrics, National Institutes of Health Clinical Center, Bethesda, MD, United States; 4 Translational Biobehavioral and Health Promotion Branch, National Institutes of Health Clinical Center, Bethesda, MD, United States

**Keywords:** CAH, CAHQL, congenital adrenal hyperplasia, health-related quality of life, patient-reported outcome, PRO

## Abstract

**Background:**

Classic congenital adrenal hyperplasia (CAH) requires lifelong glucocorticoid (GC) therapy and the knowledge of how and when to administer GC stress doses to prevent life-threatening adrenal crises. The aim of our study was to investigate patient perception of CAH self-management in relation to clinical and socioeconomic characteristics and health-related quality of life (HRQoL).

**Methods:**

Sixty-nine patients with classic CAH, aged 16 to 75 years, participating in the study to create the disease-specific patient-reported outcome tool, CAHQL, answered 7 questions about disease self-management. Patient perceptions regarding CAH self-management were analyzed in relation to clinical and socioeconomic characteristics, biochemical disease control and CAHQL domain scores (General Health, Adrenal Insufficiency, Glucocorticoid Excess, Physical Functioning, Mental Health and Cognition, Social Functioning, and Sexual Functioning).

**Results:**

Eighty percent reported that they mostly or strongly agree that they feel comfortable managing CAH whereas over half worry about adrenal crises. All CAHQL domain scores were strongly correlated with at least one self-management question (*p* < 0.05); better HRQoL was associated with greater confidence in self-managing CAH. Increased worry about stress dosing and adrenal crises was associated with greater number of stress doses and hospitalizations in the past 6 months (*p* < 0.05). Greater number of household members, being married and male sex were associated with increased confidence in receiving help from others regarding adrenal crisis (*p* < 0.05). After adjusting for relationship status, sex remained significant. No associations were found between patient perceptions regarding CAH self-management and phenotype, age, biomarkers of disease control, insurance type, income bracket, education or work status.

**Conclusion:**

Confidence regarding self-management of CAH is associated with better HRQoL as measured by CAHQL and was not related to socioeconomic characteristics or level of disease control. Integrating systematic evaluation of self-management skills and the HRQoL burden of having CAH into standard of care is recommended.

## 1 Introduction

Congenital adrenal hyperplasia (CAH) due to 21-hydroxylase deficiency is an autosomal recessive disorder characterized by impaired cortisol biosynthesis, aldosterone deficiency and androgen excess. In its classic form, CAH presents in infancy or early childhood and requires lifelong glucocorticoid (GC) therapy and usually mineralocorticoid therapy to replace deficient cortisol and aldosterone and suppress excessive adrenocorticotropic hormone (ACTH) driven androgen production (1).

The most serious risk for individuals with CAH is adrenal insufficiency during periods of physiological stress, such as illness, trauma, or surgery. Without prompt and adequate administration of stress-dose GCs, patients are at risk of adrenal crisis, a life-threatening condition characterized by hypotension, electrolyte imbalance, and possible hypoglycemia (2). Adrenal crises have been reported as the leading cause of death in patients with CAH (3, 4), and are most often triggered by infectious illnesses, especially gastrointestinal illnesses (5, 6). Despite the critical role of self-management in mitigating adrenal crisis risk, limited data is available on how individuals with CAH perceive their ability to manage their condition, particularly in relation to stress dosing. Several studies have reported that even with structured education, gaps in knowledge and confidence remain common, especially in adults (6). In addition, increased rates of adrenal crises and hospitalizations have been linked to suboptimal use of stress-dose GCs and poor adherence to emergency protocols (7).

Furthermore, while the physical and psychosocial burden of CAH is well-documented, the relationship between confidence in disease self-management and health-related quality of life (HRQoL) remains underexplored. Previous research has shown that both over- and under-treatment with GCs can negatively affect HRQoL through metabolic complications, emotional distress, and social limitations (8), yet no prior studies of patients with CAH have linked these outcomes directly to patients’ perceptions of their own self-management ability.

To address these gaps, we investigated how patients with classic CAH perceive their ability to self-manage their condition, including stress dosing during illness, and examined how these perceptions relate to clinical characteristics, socioeconomic factors, and HRQoL, using the newly developed CAH-specific patient-reported outcome tool, CAHQL (9).

## 2 Materials and methods

### 2.1 Patient-reported outcome questionnaire

We previously developed and validated a CAH-specific HRQoL patient reported outcomes (PRO) instrument, CAHQL. The CAHQL is comprised of seven domains: General Health, Adrenal Insufficiency, GC Excess, Physical Functioning, Mental Health and Cognition, Social Functioning, and Sexual Functioning (9).

In the present study, we report data collected during the development of the CAHQL instrument to assess patient-reported perceptions of self-management of CAH. The development of the CAHQL followed the Food and Drug Administration (FDA) recommendations for the construction of PRO instruments. Briefly, 12 patients with classic CAH due to 21-hydroxylase deficiency were interviewed in the initial design. The framework was then adjusted by performing cognitive interviews with 6 voluntary patients and recording qualitative responses and CAH experts. Questions were adapted from Willis et al., 2013 (10) and included cognitive probe categories of comprehension/interpretation, paraphrasing, confidence judgement, recall, specific, and general. All cognitive interviews were conducted remotely. This process resulted in the creation of a preliminary 73-item questionnaire, which was subsequently administered to the 69 cohort patients for validity and reliability testing.

Among those 73 items, a series of 7 questions assessed patients’ perceptions of CAH self-management and did not ultimately become part of the CAHQL instrument due to poor inter-domain consistency. Self-management questions used a 6-point Likert scale, ranging from “strongly disagree” (1) to “strongly agree” (6) and reverse coded when appropriate to align higher instrument scores with better quality of life (**Table 1**). CAHQL item raw scores were linearly transformed to a 0 to 100 scale, reverse coded when appropriate, and the total sum was divided by the number of domain items to obtain domain scores (9).

**Table 1:** CAHQL questions administered related to the perceptions of CAH self-management.

The following statements are about the management of CAH. Please pick the answer choice that best describes how you have felt over the past 4 weeks.						
	Strongly disagree	Mostly disagree	Somewhat disagree	Somewhat agree	Mostly agree	Strongly agree
I feel comfortable managing CAH on my own.						
People around me know how to help me if I were to go into an adrenal crisis.						
I know when to give myself stress dose steroids.						
I worry about not giving myself enough steroids in the setting of illness.						
I worry that I give myself too much steroids in the setting of illness.						
I feel comfortable giving myself the emergency hydrocortisone injection if needed.						
I worry about going into adrenal crisis.						

REDCap (Research Electronic Data Capture) was used to administer the survey electronically (11).

### 2.2 Patient cohort characteristics

Briefly, sixty-nine patients with classic CAH due to 21-hydroxylase deficiency 16 years of age or older who were being followed longitudinally in a Natural History Study at the National Institutes of Health Clinical Center in Bethesda, Maryland, USA (ClinicalTrials.gov identifier NCT00250259) were recruited to develop the CAHQL (9). Adolescents and young adults were categorized as under 26 years of age (12). Laboratory measurements were obtained within 6 months of administering the CAHQL. Fasting bloodwork was drawn in the morning prior to medication administration. This study was conducted with approval by the National Institutes of Health Institutional Review Board (IRB). All patients (if adults) or parents (for children) provided written informed consent, and patients younger than 18 years gave written assent.

Patient data collected included clinical characteristics [age, body mass index (BMI), sex, CAH phenotype, GC preparation (short- vs long-acting), and GC dose]; patient-reported treatment-related occurrences (missed doses in the past 4 weeks, number of stress doses, emergency department visits, and hospitalizations within the past 6 months); early morning prior to medication laboratory measurements (17-hydroxyprogesterone, androstenedione, and plasma renin activity); sociodemographic factors (relationship status, insurance type, education level, employment status, household size, and income bracket); and CAHQL domain scores.

Obesity was defined as having a BMI > 30 according to the Centers for Disease Control and Prevention (CDC) standards. For laboratory measurements, androstenedione and 17-hydroxyprogesterone were measured by liquid chromatography-tandem mass spectrometry at the NIH Clinical Center (Bethesda, MD). Poor CAH control was defined as androstenedione levels above age- and sex-specific normal range.

### 2.3 Statistical analyses

Descriptive statistics were performed for sociodemographic and clinical characteristics and reported as a percentage or mean ± SD. Associations between CAH self-management questions and cohort characteristics were assessed using Spearman’s rank correlation (r_s_), Wilcoxon rank sum tests, and Kruskal-Wallis tests, as per data distributional assumptions. *Post-hoc* tests with Bonferroni correction were used to identify group differences when applicable. Multiple linear regression models were used to analyze independent associations for demographic variables. Data were analyzed using R v.4.4.1 (R Foundation for Statistical Computing, Vienna, Austria). *P*-values < 0.05 were considered significant.

## 3 Results

### 3.1 Patient characteristics

Sixty-nine patients with classic congenital adrenal hyperplasia (CAH) due to 21-hydroxylase deficiency participated in the study (aged 16 to 75 years old, average age of 33.8 ± 15.0 years). The majority (66.7%) of patients had salt-wasting phenotype. Pediatric patients under the age of 18 years were evaluated every 4 to 6 months and adults were evaluated every 6 to 12 months. Patients varied in age, socioeconomic status, clinical status, and symptomology (**Table 2**).

**Table 2:** Demographics and clinical characteristics.

Characteristic	Cohort (N = 69)
Age, y	33.8 ± 15.0
Young adults (≤26y)	24 (34.8)
Salt-wasting	46 (66.7)
Sex^1^	
Male	34 (49.3)
Female	34 (49.3)
Education Level	
Partial high school	5 (7.3)
High school graduate	15 (21.7)
Partial college	10 (14.5)
College graduate	19 (27.5)
Graduate/professional training	20 (29.0)
Health Insurance	
Private	53 (76.8)
Medicare/Medicaid	9 (13.0)
Unknown	7 (10.1)
Annual income	
<$15,000	3 (4.3)
$25-50,000	6 (8.7)
$50-75,000	10 (14.5)
$75-100,000	15 (21.7)
>$100,000	28 (40.6)
Unknown	7 (10.1)
Body mass index (BMI)	29.6 ± 8.9
Obese	22 (31.9)
Height SD	-0.5 ± 1.2
Glucocorticoid treatment	
Hydrocortisone	35 (50.7)
Prednisone or prednisolone	15 (21.7)
Methylprednisolone	7 (10.1)
Dexamethasone	8 (11.6)
Combination	4 (5.8)
Missed doses per month	1.6 ± 3.1
Glucocorticoid dose^2^ (mg/m^2^/day)	16.3 ± 8.9
Glucocorticoid dose^2^ (mg/day)	31.6 ± 20.2
Relationship status	
Never married	45 (65.2)
Married or Domestic Partnership	20 (29.0)
Divorced or Widowed	4 (5.8)
Menses in last 6 months (females)	22 (64.7)
Menstrual frequency (days) (females)	29.6 ± 15.6
Desire for children	29 (42.0)
Sexually active	30 (43.5)
Male	19 (55.9)
Female	11 (32.4)
In the past 6 months:	
17-hydroxyprogesterone ng/dL^3^	4046.9 ± 5607.4
Androstenedione^4^	
Normal/Suppressed	39 (67.2)
Elevated	19 (32.8)
Plasma renin activity elevated^5^	25 (44.6)
Illness	
Individuals with illness	37 (53.6)
Number of illnesses	0.9 ± 1.1
Stress dose for illness	
Oral	26 (37.7)
Intramuscular (IM) injection	9 (13.0)
Stress dose for other reason	10 (14.5)
Number of times stress dose for other reasons	3.6 ± 2.8
Total number of stress dosing times	1.1 ± 2.0
Emergency room visit	11 (15.9)
Hospitalization	2 (2.9)

Data presented as N (%) or mean ± SD.

^1^ N = 1 transgender male excluded from analyses that included sex.

^2^ Hydrocortisone equivalent dose calculated as: prednisone/ prednisolone dose (mg) multiplied by 5, methylprednisolone (mg) by 6, and dexamethasone (mg) by 80.

^3^ N = 57 due to missing laboratory evaluation.

^4^ N = 58 due to missing laboratory evaluation.

^5^ N = 56 patients due to missing laboratory evaluation.

Nineteen patients (32.8%) were experiencing poor disease control, which was captured from the most recent labs within 6 months of administering the patient-reported outcome (PRO) survey. Half of patients (50.7%) were taking short-acting hydrocortisone and the average GC dose was 16.3 ± 8.9 mg/m^2^/day. The number of patient-reported missed GC doses per month was 1.6 ± 3.1 doses. In the past 6 months, over half of patients had an illness (37 patients, 53.6%); 35 patients (50.7%) reported stress dosing for illness, with the majority (26 patients, 37.7%) adjusting oral doses while 9 patients (13.0%) administered an intramuscular (IM) injection of Solu-Cortef. The average number of times an individual stress dosed in the past 6 months was 1.1 ± 2.0, for infectious illness or other reasons. Eleven patients (15.9%) experienced an emergency room (ER) visit while two patients were hospitalized in the past 6 months. Clinical complaints in the past 4 weeks varied and included increased salt-cravings (15.9%), weight gain (17.4%) and acne (30.4%) (**Table 3**). Ten (29.4%) women had oligomenorrhea and 11 (33.3%) men had testicular adrenal rest tissue (TART).

**Table 3:** Self-reported symptomatology in the past four weeks.

Characteristic	Cohort (N = 69)
Increased salt cravings	11 (15.9)
Acne	21 (30.4)
Excess facial hair (female)	6 (17.6)
Weight gain	12 (17.4)
New stretch marks	5 (7.2)
Change in appetite	
Decrease or no change	63 (91.3)
Increase	6 (8.7)
Sleep, hours per day	7.2 ± 1.2
Time to fall asleep (minutes)	32.3 ± 36.4
Minutes of exercise per week	197.7 ± 244.6
Work or school days lost	1.1 ± 3.9
Sexual functioning	
Vaginal dilators	3 (8.8)
Problems with libido or erections	16 (23.2)

Data presented N (%) or mean ± SD.

About half of the patients (56.5%) had at least a college level education and most (76.8%) had private health insurance with a median annual income between $75-100,000 (**Table 2**). The majority (65.2%) of patients were never married, while 42.0% of patients expressed a wish to have children, and less than half (43.5%) had been sexually active in the past 4 weeks. Among all patients, 46.0% were found to have comorbidities, with the most common being mood/mental health issues (10 patients, 14.5%) followed by attention-deficit/hyperactivity disorder (10 patients, 14.5%), and hypertension (7 patients, 10.1%). In the 6 months prior to the study, no patients were hospitalized due to mental health issues.

### 3.2 Perceptions of CAH self-management questions

Seven questions pertained to the patient perceptions of self- management of CAH and stress dosing behavior over the past 4 weeks (**Table 1**). Overall, 79.7% of patients mostly or strongly agreed that they feel comfortable managing CAH on their own, and 87.0% mostly or strongly agreed that they know when to give themselves stress steroids. However, 58.0% of patients mostly or strongly agreed that they worry about going into adrenal crisis and only 50.7% of patients mostly or strongly agreed that they feel comfortable self-administering a hydrocortisone injection if needed. Patients reported more worry about increasing steroids too much (78.3%) rather than not enough (65.2%) during illnesses.

### 3.3 Stress dosing

**Figure 1:**
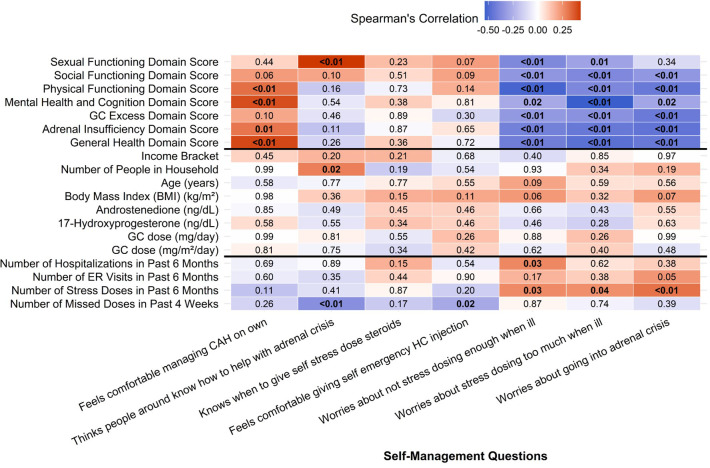
Associations between self-management perceptions, patient characteristics and health-related quality of life measured by CAHQL. CAHQL domain scores (n = 7). Red indicates positive correlations (r_s_), blue indicates negative correlations, and *p*-values are written in black text. Darker shades indicate correlation value closer to +1 or -1, respectively. Bolded values indicate significant univariate association of *p*  < 0.05.

Higher number of stress doses in the past 6 months was associated with increased worry about not giving enough (*p* = 0.03) or too much *(p* = 0.04) stress steroids during illness and going into adrenal crises (*p* < 0.01), but no difference was observed in relation to CAH phenotype (**Figure 1**). Patients with frequent hospitalizations (*p* = 0.03) and female sex (*p* = 0.04) expressed greater concern about not stress dosing enough when ill.

Furthermore, individuals who reported better compliance, reporting fewer or no missed doses in the past 4 weeks, had greater comfort giving themselves emergency hydrocortisone injection if needed (*p* = 0.02) and were more confident that people around them knew how to help them in a crisis (*p* < 0.01). Worry about not stress dosing enough and stress dosing too much were negatively associated with all CAHQL domains (*p* ≤ 0.02), and worry about going into adrenal crisis was negatively associated with all CAHQL domains (*p* ≤ 0.02) except Sexual Functioning domain (**Figure 1**).

### 3.4 Sociodemographic characteristics and hormonal measurements

Patients who had others living in their household (*p* = 0.02), were married or in a domestic partnership (*p* = 0.03), or had self-reported good compliance (*p* < 0.01) reported higher agreement with the statement “People around me know how to help me if I were to go into an adrenal crisis.” (**Figure 1**). Male sex was also associated with higher agreement with this statement compared to female sex (*p* < 0.01). After adjusting for relationship status, sex remained significant (β = -0.99, 95% CI: -1.69 - -0.30; *p* < 0.01).

No other significant associations between sociodemographic factors, and self-management questions were observed. Adolescent and young adult patient responses did not differ from adult patient responses. Similarly, no associations were found between steroid hormone biomarkers of disease control or GC preparation/dose and patient-reported self-management of CAH.

### 3.5 CAHQL domain scores in relation to perceptions of self-management

All seven CAHQL domain scores were strongly correlated with at least one self-management question (**Figure 1**). Specifically, better quality of life was associated with reduced worry about stress dosing amounts and adrenal crises, and with greater comfort of managing CAH (all *p* < 0.05).

## 4 Discussion

In our study, confidence regarding self-management of CAH was associated with higher quality of life, as evidenced by higher CAHQL scores in all 7 domains. This finding was not associated with differences in disease control, CAH phenotype, age, or socioeconomic characteristics. Patients who in the past 6 months had been hospitalized or had frequent use of stress dose steroids were more likely to worry about stress dosing and adrenal crises, especially women. Only half of the cohort reported feeling comfortable giving themselves an emergency hydrocortisone injection, and most patients worried about giving too little or too much hydrocortisone during times of illness. Worrying about stress dosing and having an adrenal crisis was associated with lower quality of life across all CAHQL domains. These findings highlight the importance of evaluating the psychosocial and behavioral factors that influence disease management and stress dosing, and the need to address the HRQoL burden of CAH in clinical settings.

Perceived difficulty with self-management has been previously linked to impaired quality of life amongst patients with primary, secondary, or GC-induced adrenal insufficiency (13, 14). Specifically, providing in-depth education on self-management and ensuring better financial and family support were identified as ways to improve HRQoL outcomes. Access to disease-specific education and social support networks through the establishment of comprehensive care centers with multidisciplinary specialist teams has been proposed to mitigate barriers in the management of CAH (15, 16). Psychosocial support has been shown to improve outcomes in a range of chronic conditions (17–19), and expert opinion has recommended that psychosocial screening be both general (i.e. psychiatric symptoms) and disease-specific (i.e. sexual distress or adrenal insufficiency) for those with CAH (17). More than half of our cohort experienced persistent worry about the possibility of an adrenal crisis, which was associated with lower HRQoL across all CAHQL domains except Sexual Functioning.

In our study, patients’ responses to the instrument’s questions did not differ by insurance type, income, education, or employment status suggesting that these concerns are widespread regardless of socioeconomic background. There is a paucity of data regarding the relationship between patient education level and the effectiveness of stress dose teaching interventions with only one study finding a significant difference exclusively between the lowest and highest education levels (20). Moreover, fear of adrenal crisis did not correlate with disease severity or level of disease control, meaning that substantial distress can be present even among those who appear to be clinically well managed. Notably, patients with classic CAH have increased prevalence of anxiety, depression, alcohol misuse, suicidality, and adjustment disorders compared with healthy controls (17, 21, 22). These mental health vulnerabilities highlight the importance of addressing emotional well-being as part of routine medical care. Pediatric and adult healthcare providers alike should be comfortable screening for psychiatric issues and referring patients to knowledgeable specialists who understand the unique psychological challenges of CAH.

Although age was not a significant factor influencing distress or self-management skills, nearly half of our cohort consisted of individuals under 30 years old, underscoring the need to support adolescents and young adults as they transition care from pediatric to adult endocrinology. Adequate transition readiness for CAH patients is linked to better medication adherence, higher quality-of-life scores, and greater likelihood of maintaining consistent follow-up with adult endocrinologists (23). Therefore, assessing preparedness for transition, reinforcement of self-management competencies, and continuity of support are vital to smoothing this vulnerable period and reducing anxiety around disease management. Several frameworks for transition to adult medicine have been proposed, showcasing structured approaches to guide this process (24–28).

Clinical factors associated with heightened worry about stress dosing appropriately and adrenal crises included recent hospitalizations and increased frequency of stress dosing in the past 6 months, with women being particularly affected. Existing evidence on patient perceptions of adrenal crises and stress dosing in primary and secondary adrenal insufficiency is largely limited to qualitative studies with semi-structured interviews. Repetitive education, optimizing social support and health care professional training have been identified as key factors in improving patient self-management of adrenal crisis prevention (20, 29, 30). Our findings highlight subgroups that deserve further attention during clinical visits and suggest that clinicians should not rely solely on clinical indicators of disease control to identify patients in need of additional support. Rather, routine clinic visits should include standardized screening for distress related to emergencies and self-management, assessment of the emotional burden of the disease, demonstration of key self-care skills, and evaluation of knowledge gaps (17). When persistent distress is identified, even in individuals who otherwise demonstrate good disease control, a referral to a qualified mental health professional is warranted (18). Early recognition of distress and targeted interventions can strengthen self-management and ultimately improve health outcomes and overall quality of life.

Nearly half of our cohort was uncomfortable with providing themselves with emergency injectable hydrocortisone, which is in agreement with previous studies of adrenal insufficiency (31, 32). Despite the fact that most of our patients carried an emergency hydrocortisone kit and had received appropriate and repeated training, low confidence with injection techniques persisted. Knowing when to stress dose and feeling comfortable self-injecting was not associated with any HRQoL measure, clinical characteristic or level of disease control except increased self-reported compliance with medication regimen. In an on-line survey distributed through adrenal insufficiency patient advocacy groups, multiple barriers were identified as playing a role in patients’ hesitancy to self-inject including: the complexity of the multi-step process of hydrocortisone injection, injection-related anxiety, and equipment challenges (31). These factors may have contributed to our findings as we did not evaluate possible underlying causes of injection hesitancy.

In our study, relationship status and household composition were strongly associated with patients’ perceptions that those around them know how to respond in the event of an adrenal crisis. Specifically, individuals who were married or lived with others were more likely to report confidence that other people could provide appropriate support during an emergency. This finding underscores the critical role of social support networks in perceived safety and preparedness for acute events, which may improve adherence to stress dosing protocols and quality of life. Increased mortality in patients with CAH due to adrenal crises has been reported (3) and family education of adrenal insufficiency management has been highlighted as a key preventative strategy (33). Ensuring that patients and their household members are trained in emergency hydrocortisone administration is essential. Clinicians should routinely assess patients’ social environment and provide targeted education and training to strengthen emergency preparedness, particularly for those with limited support networks or those who are transitioning to adult care.

Our study was based on a robust assessment of factors influencing quality of life and disease self-management, drawing on aggregated data from cognitive interviews and expert input, initially conducted to inform a disease-specific HRQoL questionnaire. HRQoL was measured using CAHQL, a validated instrument tailored to this patient population. Evidence from chronic disease populations demonstrates that patient perceptions are important determinants of self-management behaviors (34–36). However, these relationships are often indirect and, while they may be reflected in patient-reported outcomes and HRQoL surveys, they do not consistently translate into improvements in objective clinical endpoints. In addition, the cross-sectional design of the study limits causal inference, and HRQoL is both influenced by and has a direct impact on patients’ perceived confidence in self-management behavior. Although our study population captured a broad range of ages, clinical characteristics, and disease control, most participants received care at the NIH as part of a Natural History Study at no cost, potentially limiting the observed impact of socioeconomic stressors. Moreover, although the perceptions of self-management question list used for this study was developed from a conceptual framework specifically designed for patients with CAH and may serve as a useful screening guide, it lacks internal consistency and cannot be used as a tool to assess management skills or emotional distress in relation to HRQoL. However, administration of CAHQL, a validated PRO instrument designed to capture CAH-specific HRQoL outcomes, including both physical and mental well-being, could be used to identify at-risk patients and can be administered in less than 10 minutes.

In summary, our findings highlight that confidence in self-management of CAH is linked to better quality of life, irrespective of disease control, phenotype, age, or socioeconomic factors, while persistent anxiety about adrenal crises remains common. Social support, particularly through marital status or larger households, enhances patients’ perceived preparedness for emergencies, underscoring the importance of involving family or cohabitants in education and training. Competence with stress dosing is critical, given the high risk of mortality from adrenal crises. Standardized assessment of self-management skills, emotional burden, and knowledge gaps should be integrated into routine clinical care of patients with CAH. Transition readiness in adolescents and young adults further supports adherence, quality of life, and continuity of care with adult endocrinology providers. Overall, our study underlines the need for a comprehensive, patient-centered care model that addresses both the medical and psychosocial aspects of CAH. Administering CAHQL is a useful tool for identifying patients at risk of lacking self-management skills and in need of more comprehensive care.

## Data Availability

The raw data supporting the conclusions of this article will be made available by the authors, without undue reservation.

## References

[1] Merke DP Auchus RJ . Congenital adrenal hyperplasia due to 21-hydroxylase deficiency. N Engl J Med. (2020) 383:1248–61. doi: 10.1530/ey.18.8.16. PMID: 32966723

[2] Mallappa A Merke DP . Management challenges and therapeutic advances in congenital adrenal hyperplasia. Nat Rev Endocrinol. (2022) 18:337–52. doi: 10.1530/ey.19.8.16. PMID: 35411073 PMC8999997

[3] Falhammar H Frisén L Norrby C Hirschberg AL Almqvist C Nordenskjöld A . Increased mortality in patients with congenital adrenal hyperplasia due to 21-hydroxylase deficiency. J Clin Endocrinol Metab. (2014) 99:E2715–21. doi: 10.1210/jc.2014-2957. PMID: 25279502

[4] Jenkins-Jones S Parviainen L Porter J Withe M Whitaker MJ Holden SE . Poor compliance and increased mortality, depression and healthcare costs in patients with congenital adrenal hyperplasia. Eur J Endocrinol. (2018) 178:309–20. doi: 10.1530/eje-17-0895. PMID: 29371334

[5] Reisch N Willige M Kohn D Schwarz HP Allolio B Reincke M . Frequency and causes of adrenal crises over lifetime in patients with 21-hydroxylase deficiency. Eur J Endocrinol. (2012) 167:35–42. doi: 10.1530/eje-12-0161. PMID: 22513882

[6] El-Maouche D Hargreaves CJ Sinaii N Mallappa A Veeraraghavan P Merke DP . Longitudinal assessment of illnesses, stress dosing, and illness sequelae in patients with congenital adrenal hyperplasia. J Clin Endocrinol Metab. (2018) 103:2336–45. doi: 10.1210/jc.2018-00208. PMID: 29584889 PMC6276663

[7] Tschaidse L Wimmer S Nowotny HF Auer MK . Frequency of stress dosing and adrenal crisis in paediatric and adult patients with congenital adrenal hyperplasia: a prospective study. Eur J Endocrinol. (2024) 190:275–83. doi: 10.1093/ejendo/lvae023. PMID: 38584334

[8] Falhammar H Nyström HF Thorén M . Quality of life, social situation, and sexual satisfaction, in adult males with congenital adrenal hyperplasia. Endocrine. (2014) 47:299–307. doi: 10.1007/s12020-013-0161-2. PMID: 24408051

[9] Flokas ME Yang L Middleton KR Kollender S Parker M Sukin C . CAHQL: A patient-reported outcome instrument to assess health-related quality of life in congenital adrenal hyperplasia. (2025) 110:e1149–59. doi: 10.1210/clinem/dgae309 PMC12168074 38706369

[10] Willis GB Artino AR . What do our respondents think we're asking? Using cognitive interviewing to improve medical education surveys. J Grad Med Educ. (2013) 5:353–6. doi: 10.4300/jgme-d-13-00154.1. PMID: 24404294 PMC3771159

[11] Harris PA Taylor R Thielke R Payne J Gonzalez N Conde JG . Research electronic data capture (REDCap)--a metadata-driven methodology and workflow process for providing translational research informatics support. J BioMed Inf. (2009) 42:377–81. doi: 10.1016/j.jbi.2008.08.010. PMID: 18929686 PMC2700030

[12] Lestishock L Cuomo C Hickam T Johnson-Hooper T Maddux M Muzzall E . Self-perceived importance and confidence of adolescents transitioning to adult care. Health Care Transit. (2025) 3:100086. doi: 10.1016/j.hctj.2024.100086 39712478 PMC11657790

[13] Li D Genere N Behnken E Xhikola M Abbondanza T Vaidya A . Determinants of self-reported health outcomes in adrenal insufficiency: a multisite survey study. J Clin Endocrinol Metab. (2021) 106:e1408–19. doi: 10.1210/clinem/dgaa668. PMID: 32995875 PMC7947833

[14] Li D Brand S Hamidi O . Quality of life and its determinants in patients with adrenal insufficiency: a survey study at 3 centers in the United States. (2022) 107:e2851–61. doi: 10.1210/clinem/dgac175 PMC9202727 35350067

[15] Eitel KB Fechner PY . Barriers to the management of classic congenital adrenal hyperplasia due to 21-hydroxylase deficiency. J Clin Endocrinol Metab. (2025) 110:S67–73. doi: 10.1210/clinem/dgae710. PMID: 39836619 PMC11749880

[16] Sandberg DE Gardner M Lapham ZK . Mental health issues associated with classic congenital adrenal hyperplasia due to 21-hydroxylase deficiency. J Clin Endocrinol Metab. (2025) 110:S46–55. doi: 10.1210/clinem/dgae668. PMID: 39836615 PMC11749910

[17] Claahsen-van der Grinten HL Speiser PW . Congenital adrenal hyperplasia-current insights in pathophysiology, diagnostics, and management. (2022) 43:91–159. doi: 10.1210/endrev/bnab016 PMC8755999 33961029

[18] Young-Hyman D de Groot M Hill-Briggs F Gonzalez JS Hood K Peyrot M . Psychosocial care for people with diabetes: a position statement of the American Diabetes Association. Diabetes Care. (2016) 39:2126–40. doi: 10.2337/9781580404396 PMC5127231 27879358

[19] Simpson A Ross R Porter J Dixon S Whitaker MJ Hunter A . Adrenal insufficiency in young children: a mixed methods study of parents' experiences. J Genet Couns. (2018) 27:1447–58. doi: 10.1136/bmj.1.4668.1473 PMC6209050 29982889

[20] van der Meij NT van Leeuwaarde RS Vervoort SC Zelissen PM . Self-management support in patients with adrenal insufficiency. Clin Endocrinol. (2016) 85:652–9. doi: 10.1111/cen.13083. PMID: 27063934

[21] Engberg H Butwicka A Nordenstrom A Hirschberg AL Falhammar H Lichtenstein P . Congenital adrenal hyperplasia and risk for psychiatric disorders in girls and women born between 1915 and 2010: a total population study. Psychoneuroendocrinology. (2015) 60:195–205. doi: 10.1016/j.psyneuen.2015.06.017. PMID: 26184920

[22] Harasymiw LA Grosse SD Cullen KR Bitsko RH Perou R Sarafoglou K . Depressive and anxiety disorders and antidepressant prescriptions among insured children and young adults with congenital adrenal hyperplasia in the United States. Front Endocrinol (Lausanne). (2023) 14:1129584. doi: 10.3389/fendo.2023.1129584. PMID: 37664854 PMC10470620

[23] Ekbom K Lajic S Falhammar H Nordenström A . Transition readiness in adolescents and young adults living with congenital adrenal hyperplasia. Endocrine Practice: Off J Am Coll Endocrinol Am Assoc Clin Endocrinol. (2023) 29:266–71. doi: 10.1016/j.eprac.2023.01.010. PMID: 36693541

[24] Bachelot A . Transition of care from childhood to adulthood: congenital adrenal hyperplasia. Endocr Dev. (2018) 33:17–33. doi: 10.1159/000487523. PMID: 29886487

[25] Twito O Shatzman-Steuerman R Dror N Nabriski D Eliakim A . The "combined team" transition clinic model in endocrinology results in high adherence rates and patient satisfaction. J Pediatr Endocrinol Metab. (2019) 32:505–11. doi: 10.1515/jpem-2019-0056. PMID: 31028713

[26] Le Roux E Menesguen F Tejedor I Popelier M Halbron M Faucher P . Transition of young adults with endocrine and metabolic diseases: the 'TRANSEND' cohort. Endocr Conn. (2021) 10:21–8. doi: 10.1530/ec-20-0520. PMID: 33263561 PMC7923139

[27] Pitts L Armstrong A Fleming L Hallowell S Landier W Odom JN . A scoping review of literature exploring the healthcare transition of individuals with congenital adrenal hyperplasia. Horm Res Paediatr. (2025) 1–10. doi: 10.1159/000547790 40763708

[28] Claahsen-van der Grinten HL Davidse K Dreijerink KMA Eck JPV Reisch N Lajic S . Enhancing transition care for adolescents and young adults with adrenal insufficiency in the Netherlands: a holistic model for improved patient outcomes. Endocr Conn. (2025) 14. doi: 10.1530/ec-25-0324. PMID: 40960017 PMC12508302

[29] Chua A Yoeli H Till D Dashora U Oyibo P Drake WM . Factors influencing self-management of adrenal crisis in patients with adrenal insufficiency: a qualitative study. Endocr Connect. (2025) 14(5):e240651. doi: 10.1530/EC-24-0651. PMID: 40214077 PMC12060673

[30] Ross IL Llahana S Anderson MM Demeke B Minnie EM Wass JAH . Identifying knowledge gaps in individuals with primary adrenal insufficiency: a critical step in preventing adrenal crisis. Clin Endocrinol. (2025) 103:659–68. doi: 10.1111/cen.70006. PMID: 40696758 PMC12492782

[31] Llahana S Anthony J Sarafoglou K Geffner ME Ross R . Patient and caregiver experiences with hydrocortisone injections in adrenal crisis: a mixed-methods cross-sectional study. Front Endocrinol (Lausanne). (2025) 16:1544502. doi: 10.3389/fendo.2025.1544502. PMID: 40331138 PMC12053486

[32] Hover WJ Krein AD Kallet J Kinney GL Speiser PW Witchel SF . People with adrenal insufficiency who are in adrenal crisis are frequently unable to self-administer rescue injections. Endocrine Practice: Off J Am Coll Endocrinol Am Assoc Clin Endocrinol. (2025) 31:625–30. doi: 10.1016/j.eprac.2025.02.017. PMID: 40043845

[33] Camtosun E Sangun O . Treatment and prevention of adrenal crisis and family education. J Clin Res Pediatr Endocrinol. (2025) 17:80–92. doi: 10.4274/jcrpe.galenos.2024.2024-6-12-S 39713905 PMC11730097

[34] Tan F Oka P Dambha-Miller H Tan NC . The association between self-efficacy and self-care in essential hypertension: a systematic review. BMC Fam Pract. (2021) 22:44. doi: 10.1186/s12875-021-01391-2. PMID: 33618661 PMC7901221

[35] Magi CE El Aoufy K Amato C Longobucco Y Bambi S Vellone E . The association between self-care and health literacy in patients with chronic diseases: a systematic review and meta-analysis. J Clin Nurs. (2026). doi: 10.1111/jocn.70291. PMID: 41854032

[36] Bailey A Mallow J Theeke L . Perceived self-efficacy, confidence, and skill among factors of adult patient participation in transitional care: a systematic review of quantitative studies. SAGE Open Nurs. (2022) 8:23779608221074658. doi: 10.1177/23779608221074658. PMID: 35111928 PMC8801722

